# Autophagy in Thyroid Cancer: Present Knowledge and Future Perspectives

**DOI:** 10.3389/fendo.2015.00022

**Published:** 2015-02-18

**Authors:** Romana T. Netea-Maier, Viola Klück, Theo S. Plantinga, Johannes W. A. Smit

**Affiliations:** ^1^Department of Medicine, Division of Endocrinology, Radboud University Nijmegen Medical Center, Nijmegen, Netherlands

**Keywords:** autophagy, thyroid, carcinoma, therapy, pathogenesis

## Abstract

Thyroid cancer is the most common endocrine malignancy. Despite having a good prognosis in the majority of cases, when the tumor is dedifferentiated it does no longer respond to conventional treatment with radioactive iodine, the prognosis worsens significantly. Treatment options for advanced, dedifferentiated disease are limited and do not cure the disease. Autophagy, a process of self-digestion in which damaged molecules or organelles are degraded and recycled, has emerged as an important player in the pathogenesis of different diseases, including cancer. The role of autophagy in thyroid cancer pathogenesis is not yet elucidated. However, the available data indicate that autophagy is involved in several steps of thyroid tumor initiation and progression as well as in therapy resistance and therefore could be exploited for therapeutic applications. The present review summarizes the most recent data on the role of autophagy in the pathogenesis of thyroid cancer and we will provide a perspective on how this process can be targeted for potential therapeutic approaches and could be further explored in the context of multimodality treatment in cancer and personalized medicine.

## Introduction

Thyroid carcinoma (TC) is the most common endocrine malignancy accounting for >90% of malignancies of endocrine glands (1). Worldwide, the incidence of TC has increased, which can be explained by the more intensive use of imaging modalities that increased the detection of small, non-symptomatic tumors (2). Differentiated thyroid carcinoma (DTC), which is the most prevalent TC, has an excellent 10-year prognosis of around 85% (3). Effective treatment in most DTC patients consists of total thyroidectomy followed by therapy with radioactive iodide (RAI), which is based on the unique ability of follicular thyroid cells to take up iodine via the sodium-iodide symporter protein (NIS) (4). Although prognosis of DTC is favorable, unfortunately, in a high proportion of patients with metastases, the capacity for RAI accumulation is diminished or lost.

## Thyroid Cancer Pathology and Molecular Pathogenesis

Thyroid carcinomas are categorized into three groups. DTC (97%) and anaplastic thyroid carcinoma (ATC) (1%) originate from follicular epithelial cells, whereas medullary thyroid carcinoma (2%) (MTC) originates from neuroendocrine parafollicular cells. DTC can be distinguished in many histological subtypes, major groups being papillary thyroid carcinoma (PTC), which is the most common type of TC (87%), and follicular thyroid carcinoma (FTC) (6%). Hürthle cell carcinomas (3–4%) form a distinct subgroup of DTC that are mostly FTC but can also be PTC. Mixed FTC and PTC also occur.

The genetic events involved in DTC include several gene rearrangements and gene mutations. As a consequence of these genetic mutations and/or rearrangements, signaling pathways are activated in favor of cell growth, survival, and angiogenesis. PTC frequently harbor point mutations of BRAF (30–69%) and rearrangements in the RET oncogene (40–70%) leading to MAP-kinase pathway activation (5–8). To a lesser extent, RAS gene mutations also occur in PTC (10–20%). FTC frequently harbor RAS mutations (18–52%) and/or PPARγ/PAX8 rearrangements (25–56%) (9). Mutations of PTEN may also be present in FTC. RAS mutations constitutively activate both MAPK and PI3K/AKT signaling pathways. Genetic alterations involving the PI3K/AKT signaling pathway are rare in DTC. In ATC, genetic alterations in the PI3K/AKT signaling pathway may be present (10–12). Also, mutations of the p53 tumor suppressor gene (13) are involved. Recently, mutations in the β-catenin gene have been described in TC (13–16).

## Dedifferentiation

Differentiated thyroid carcinoma initially retains the biological properties of normal thyroid cells [iodide uptake by NIS, thyroglobulin (Tg) synthesis, expression of thyroid peroxidase (TPO), and receptor for thyrotropin (TSHR) (17–19)]. However, NIS, TPO, Tg, and TSHR expression gradually decrease in DTC with NIS and TPO disappearing at an earlier stage than others (20–23). Most studies showed reduction or even absence of NIS mRNA in TCs (24–26), while immunohistochemistry studies have demonstrated that NIS protein is overexpressed in many thyroid tumors and is predominantly located in the cytoplasm (17, 21, 24). TSHR expression is persistent in nearly all DTC (21, 24, 27–29). Tg production remains preserved even in absence of iodide uptake. In ATC, Tg secretion may decrease and is eventually lost (21, 24, 27, 29). A causal relationship between genetic alterations and loss of functional NIS expression is present: tumor cells harboring BRAFV600E mutation have decreased NIS, TPO, and TSHR gene expression compared to other tumor cells without this mutation (24, 30–34). In addition, a relationship between activation of PI3K and mammalian target of rapamycin (mTOR) and loss of NIS expression has also been described (35).

## Initial Therapy

According to the consensus of the American Thyroid Association (ATA) (36, 37), with the exception of small unilateral PTC, most patients with DTC should be subjected to initial therapy consisting of near-total thyroidectomy followed by RAI remnant ablation therapy. In recent years, the benefits of RAI ablation therapy are debated and a strategy based on risk of recurrence is recommended (37). Recent landmark studies (ESTAMIBL and HiLo) have indicated that 37 mCi is as efficacious as 100 mCi for thyroid remnant ablation (37–39).

## Recurrent or Persistent Disease

When a malignant lesion is accessible, surgery can be performed. If a malignant lesion is present, which cannot be cured by surgery, RAI therapy may be performed. The remission rate in pulmonary metastases treated with RAI varies from 90% in patients with microscopic metastases to 10% in macroscopic metastatic disease and from 20 to 7% in bone metastases (37, 40, 41). Palliative therapies include external irradiation, radiofrequency ablation, chemoembolization and for pulmonary metastases, endobronchial laser therapy (42–45). Approximately 5–15% of DTC patients will develop or present with local recurrent disease or metastasis. In 25–66% of these patients, the susceptibility to RAI is diminished or lost (3, 37, 46).

Treatment options for patients with RAI resistance were until recently limited to palliative treatment, as conventional chemotherapy is not effective in DTC. Recently, novel treatment options have become available in terms of kinase inhibitors (KIs).

The DECISION trial, with sorafenib, was the first phase III study with a kinase inhibitor in DTC (46) and showed a reduction in progression free survival (PFS) of 5 months. A phase III study with lenvatinib (SELECT study) showed a reduction in PFS of 14.7 months (47). In addition to multi-kinase inhibitors (MKI), compounds have been developed the selectively target mutated kinases. Both studies have shown for the first time that MKI have a benefit on PFS in patients with advanced DTC. However, these drugs are only effective in subgroups of patients and most effects are temporary. Therefore, additional approaches are warranted. Study of pathogenetic mechanisms in DTC may reveal novel targets for therapy. Autophagy has emerged during the last years as an important player in the pathogenesis of TC.

## Autophagy and Cancer

An increasing body of literature supports the central role of autophagy in cancer development from initiation to progression and metastasis, including TC. Autophagy (“self-digestion”) is an evolutionarily conserved mechanism present in all eukaryotic cells, essential for maintaining cell homeostasis and adaptation to various stress situations including metabolic and oxidative stress resulting from nutrient deprivation or hypoxia (48). In this process, in conditions of cellular stress often resulting in excessive production of damaged proteins or organelles and reactive oxygen species (ROS), a double layer membrane is generated in the cytoplasm that engulfs defective or toxic molecules and organelles to form an autophagosome. After fusion with the lysosomes and release of lysosomal acid enzymes in the vesicle, these molecules undergo enzymatic digestion and the resulting products are either removed from the cells or are made available for synthesis of new proteins or organelles. This process is also known as macroautophagy (henceforth referred to as “autophagy”). In addition to macroautophagy, microautophagy has also been described, referring to the process of self-digestion in which no autophagosome is formed and the molecules compelled for degradation are engulfed by the lysosomes through invagination of lysosomal membrane. Furthermore, soluble proteins can be shuttled into the lysosome via the lysosomal chaperone proteins, a process termed chaperone-mediated autophagy (48).

**Figure 1:**
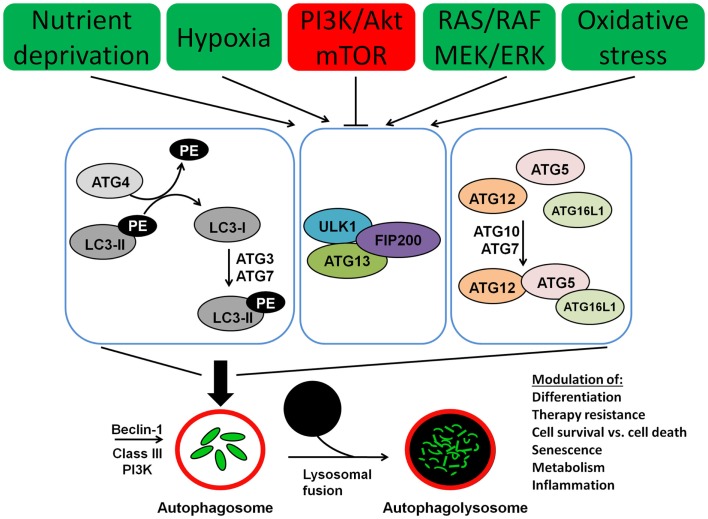
**Molecular machinery of autophagosome assembly and lysosomal fusion**. Beclin 1 is part of the type III PI3K complex that initiates autophagosome formation. Two ubiquitin-like systems are required for formation of the isolation membrane; (I) coupling of LC3 (ATG8) to phosphatidylethanolamine (PE) and (II) conjugation of ATG12 to ATG5 and ATG16L1. The five C-terminal amino acids of LC3 (unconjugated LC3-I) are cleaved of by ATG4, which is required to link the protein after activation by ATG7 and ligation by ATG3 to PE in the autophagosomal membrane (conjugated LC3-II). Similarly, ATG7 and ATG10 couple ATG12 to ATG5 and ATG16L1. This complex then localizes to the outer membrane of the forming autophagosome. Autophagosomes then fuse with lysosomes for degradation of their cargo.

Autophagy involves several Atg (autophagy) proteins, encoded by ATG genes. The function and the structure of these proteins have been initially and extensively studied in yeast species (49). However, for the majority of these proteins human orthologs have been found as well. A schematic representation of the process of autophagy is presented in Figure 1. The regulation of autophagy is very complex and not completely understood. One of the central molecules involved in the regulation of autophagy is the mTOR, which is a very potent inhibitor of autophagy (50). Moreover mTOR is involved in a complex network of signaling pathways that control and link a number of other fundamental cellular processes including cell metabolism, differentiation, proliferation, and cell survival (51, 52). The mTOR kinase activates multiple pathways that favor cellular proliferation and inhibits the autophagy pathway, thereby preventing cell cycle arrest. Furthermore, mTOR induces a metabolic shift toward glycolysis in both aerobic and anaerobic microenvironments, also known as the Warburg effect, to enable proliferation at higher rates and in hypoxic conditions, respectively (53).

Several studies link autophagy to cancer. It has been shown that autophagy can have either a positive or a negative effect on cancer development and progression depending on tumor type and stage of the disease. This apparently dual position is not completely understood but it seems to reflect the complex effect of autophagy on several fundamental processes such as cell survival, cell death, senescence, cellular metabolism, and inflammation in the context of continuous external changes in the tumor microenvironment. The role of autophagy in TC is largely unexplored. The evidence is only scarce and fragmented. Nevertheless, there are clear indications that autophagy is involved in several steps of thyroid tumorigenesis and progression as well as in therapy resistance and therefore could be exploited for therapeutic applications. In the present review, we will summarize the most recent data on the role of autophagy in the pathogenesis of TC and we will provide a perspective on how this process can be targeted for potential therapeutic approaches in TC.

## Autophagy and Tumor Initiation

In basal conditions, autophagy is fundamentally involved in maintaining the cellular homeostasis. In pathological conditions inducing cellular stress, autophagy is upregulated primarily for cytoprotective purposes. In this case, incapacity of the cells to upregulate autophagy, for instance, due to defective ATG genes, can result in accumulation of defective molecules or excessive ROS, which impair the function of cells and DNA stability resulting in disease (54).

Several mechanisms have been suggested through which autophagy might exert its tumor suppressive effects. Autophagy has a crucial role in maintaining DNA stability. As a result of oncogene activation, defective organelles produce high amounts of ROS, which when accumulated in large amounts induce oxidative stress and contribute to additional DNA damage. Autophagy represents the major buffer mechanism activated to counteract this excessive ROS production by removal of defective organelles such as mitochondria as a main source of excessive ROS through a process of selective mitophagy. In addition, autophagy contributes to the quality control of the protein synthesis. Related to this, autophagy has an important role in the clearance of defective p62, a ubiquitin-binding protein that is produced in response to metabolic stress. It has been shown that tumor cells in which the autophagy mechanism is defective accumulate excessive amounts of p62, sufficient to cause dysregulations of NF-kB signaling, accumulation of ROS and DNA damage (55). Several studies suggest that in the early phases of cancer development autophagy fulfills primarily a tumor suppressive role.

Beclin 1, encoded by the BECL1 gene located on chromosome 17q21, is a key molecule involved in the initiation of autophagy and phagophore formation. Previous studies have shown that in autophagy-deficient mice due to monoallelic deletion of BECL1 develop spontaneously malignant tumors (56). Therefore, BECL 1 is considered as a haplo-insufficient tumor suppressor gene. Moreover, somatic monoallelic deletions of BECL1 stimulate development of Hepatitis-B-induced premalignant liver lesions (56). In addition to that, monoallelic deletions of BECL1 have been found in a significant percentage of human cancers, including breast cancer, melanoma, ovary, and prostate (57–60). Furthermore, an increased Beclin 1 expression has been associated with a favorable prognosis in several cancers (60–62). In TC, however, Li et al. have found by immunohistochemistry in samples from 86 patients with PTC (57 with lymph node metastases) that Beclin 1 expression was increased in 88% of the PTC tissue samples and in 98% metastatic lymph nodes whereas in the paired normal thyroid tissues and lymph nodes without metastases from the same patients the expression of Beclin 1 was only low or absent (63). The authors suggest that Beclin 1 neo-expression correlate with the tumorigenesis and lymph node metastasis in TC. However, the mutation status of the tumors is not mentioned in this study though may be relevant for the interpretation of these data. It is known that up to 75% of PTCs carry somatic mutations in the BRAF gene, in particular the V600E BRAF (5–8). Presence of BRAF mutations has also been associated with an increased risk for lymph node metastasis in PTC. Furthermore, it has been shown that in melanoma, BRAF mutations induce autophagy (64). Therefore, whether the observed increased expression of Beclin1 and possibly the activation of autophagy in the study of Li et al. is the cause or the consequence of the malignant process, it is not clear. In addition, it has to be mentioned that the function of Beclin 1 is complex and reaches beyond autophagy, including the link between autophagy and apoptosis (65, 66). Therefore, although the exact role of Beclin1 in the development of TC is not known, these data suggest that autophagy is involved in the pathogenesis of TC.

Besides Beclin1, other autophagy-related proteins such as Atg4c, Atg5, and Atg7 also were found to have tumor suppressor functions in mice models and mutations autophagy-related genes such as ATG2B, ATG5, ATG9B, ATG 12, and UVRAG have been described in gastrointestinal cancers as well (67, 68). Furthermore, mice lacking Parkin, which is important for the ubiquitination of proteins on mitochondria contributing to the initiation of selective autophagy of damaged mitochondria, show a pro-carcinogenic phenotype (69, 70). With respect to TC, a recent study suggests that patients carrying the ATG5 single nucleotide polymorphisms rs2245214 have a higher probability to develop TC. However, no other ATG genetic variants investigated in this study showed a significant association with TC susceptibility. Furthermore, none of the selected genetic variants were associated with clinical parameters of disease progression and outcome (71).

Altogether, these studies support in part a tumor suppressive role of autophagy at least in some tumor types. In this setting, one might speculate that pharmacological induction of autophagy at least in some autophagy-deficient cells might be beneficial in the early stages of carcinogenesis. Nevertheless, none of the previous studies has established a clear causal relationship between deficient autophagy and tumorigenesis. Interestingly, other autophagy-deficient mouse models, such as the Ulk1- or Nix-deficient mice have not been shown to have an increased risk of spontaneous malignancies (72, 73). Others, such as the FIP200-deficient mice that lack an important autophagy gene have an impaired tumorigenesis (74). Moreover, Beclin1 deficiency is often associated with p53 deficiency suggesting that the function of these two proteins is interrelated (59). Therefore, although autophagy may play an important role in tumor initiation, this is not the only mechanism involved in this process and its effect probably depends on the integrity and activation of other biological processes as well, such as apoptosis.

**Figure 2:**
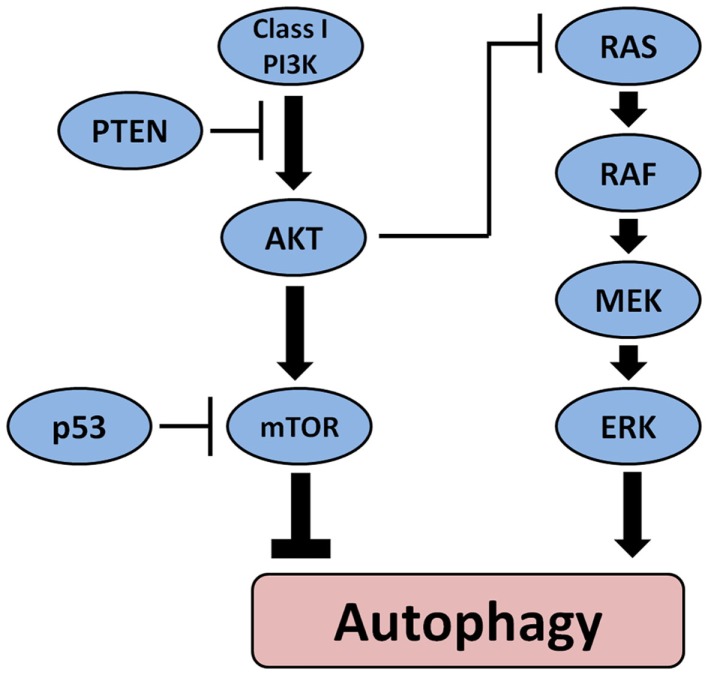
**The main pathways involved in the pathogenesis of thyroid cancer and their effect on autophagy**. Oncogenes such as Akt and class I PI3K inhibit autophagy whereas tumor suppressor PTEN and p53 can stimulate autophagy. BRAF mutations that can be found in the majority of PTC activate autophagy.

Another mechanism through which autophagy may be involved in tumorigenesis involves its regulation by other oncogenes and tumor suppressor genes. Figure 2 depicts the main pathways involved in thyroid carcinogenesis and their link with autophagy. Oncogenes such as Akt and class I PI3K inhibit autophagy whereas tumor suppressor genes such as PTEN and p53 can stimulate autophagy. Interestingly, germ-line mutations in PTEN cause a syndrome characterized by increased risk to develop malignant tumors including follicular thyroid neoplasia and FTC (75). Several somatic mutations that play an important role in thyroid cell malignant transformation can influence autophagy either positively or negatively. BRAF mutations that can be found in the majority of PTC are known to activate autophagy in melanoma (76) but possibly also in PTC (77). On the other hand, activation of PI3K/Akt/mTOR pathway (78), which has been found to be more common in ATC than in DTC (79) as well as in more aggressive tumors (80, 81), sometimes in combination with BRAF or RAS mutations (79) is associated with inhibition of autophagy. Also, inactivating mutations in the PTEN tumor suppressor gene leading to activation of the PI3K–Akt pathways have been found more often in advanced TC (82). This data suggest that in TC, they may represent late events resulting in progression to more aggressive forms. Through its position at the crossroads between the MAPK and the PI3K/Akt/mTOR pathway, RAS can play a dual role in the regulation of autophagy. It can either stimulate autophagy when the MAPK is selectively activated or inhibit autophagy through activation of PI3K pathway. Therefore, when the autophagy machinery is intact, the role of autophagy as a tumor suppressor or tumor promotor may depend apart from the histological tumor type also on the presence of certain mutations in oncogenes or tumor suppressive genes and/or the accumulation of mutations in the tumor during cancer development and progression.

## Autophagy and Tumor Progression

Once the carcinogenic phenotype has been established, autophagy plays a role in maintaining the malignant phenotype and in tumor progression through its effect on cell metabolism, cell survival, and cell death on one hand, and on the immune response in the tumor microenvironment on the other hand. In other words, the role of autophagy in tumor progression is largely dependent on the crosstalk between autophagy and other fundamental biological processes of apoptosis, senescence, immune response, and inflammation. The rapidly multiplying tumor cells rely on autophagy to maintain an increased proliferation rate in an unfavorable microenvironment characterized by hypoxia, insufficient nutrients delivery, and oxidative stress. As a result of these unfavorable conditions or as a direct effect of the oncogenic mutations, tumor cells switch for their energy metabolism to aerobic glycolysis (Warburg effect). Interestingly, as shown above, the same mutations, such as PI3k/Akt/mTOR, also inhibit the autophagy. In the context of restrictive nutrients delivery, this deficient autophagy can result in apoptotic-deficient tumor cells in increased cell death through necrosis due to the fact that the cancer cells are unable to meet the increased metabolic energy demands (83). In contrast to apoptotic cells, cells that die through necrosis, through ruptures of their plasma membrane, release several proinflammatory molecules, the damage-associated molecular patterns (DAMPs), such as uric acid, ATP/UTP, high mobility group B1 (HMGB1), in their microenvironment. These molecules help recruit and activate macrophages and other antigen presenting cells, such as dendritic cells (DC), and trigger adaptive immune responses and cytokine production that create a pro-tumorigenic environment in the peritumoral stroma, which can result in angiogenesis, tumor progression, and metastasis. In addition, both antigen presentation and antigen recognition that represent the cornerstone of this immune response are dependent on the integrity of the autophagy machinery. In this respect, it has been shown that autophagy facilitates antigen presentation by tumor cells, such as human embryonic kidney cells (HEK293T) and melanoma cells, thereby facilitating the recognition of these cells by the immune system, facilitating the removal of these cells (84). Another mechanism through which autophagy, through crosstalk with inflammation, can contribute to tumor progression is by facilitating the cellular senescence. In this process, senescent cells that are characterized by a sustained cell arrest produce cytokines that promote recruitment of immune cells that are ultimately responsible for the removal of the senescent cells but also can facilitate the tumor progression (85). Therefore, in addition to its role in maintaining the metabolic homeostasis, cellular integrity and function, and in cell survival and cell death, autophagy contributes to shaping the immune response in the tumor microenvironment.

The tumor necrosis factor (TNF) family comprises several ligands, among which TNF-related apoptosis-inducing ligand (TRAIL), which trigger apoptosis and has been shown to potently and selectively kill TC cells (86). Investigation of TRAIL has attracted attention in cancer therapy studies because they contribute to the immune cell-mediated cytotoxicity and because recombinant ligands are available for pharmacological use. Nonetheless, treatment with TRAIL has been hampered with significant therapy resistance in several cancers, including TC. It has been shown that T-helper 1-type cytokines, such as interferon (IFN)-gamma, TNF-alpha, and IL-1 beta increase the sensitivity of both normal and neoplastic thyrocytes to TRAIL. However, IGF-I and other growth/survival factors produced in the local tumor microenvironment activate the PI3K/Akt pathway, exert an anti-apoptotic effect, and are also known to inhibit autophagy through mTOR (86, 87). Recently, Jin et al. has shown that in PTC cell line TPC-1 harboring RET/PTC1 rearrangement, TRIAL treatment induced activation of autophagy and apoptosis whereas blocking autophagy using ATG7 siRNA abrogated this effect (88). In the same study, in the ATC cell line FRO, treatment with TRAIL did not induce the autophagy and blocking the autophagy with ATG siRNA sensitized FRO cells to TRAIL-induced apoptosis. Therefore, both the PTC and the ATC might benefit from modulation of autophagy. However, depending on the type of tumor and the mutation status of the tumor, either stimulation or inhibition of autophagy may be beneficial.

To add to the complexity of the role of autophagy in cell death, it has been shown that despite its primary cytoprotective functions in response to various forms of stress in normal cells, when activated excessively, it is believed that autophagy can contribute to cell death. In this case, it has been suggested that, uncontrolled excessive autophagy, such as results from overexpression of Beclin1 in mammalian cells (89), might result in cell death through either stimulation of apoptosis (90) or massive uncontrolled, possibly selective, self-digestion of cytoplasmic components beyond repair that render the cell unviable (91). Clearly, several arguments support the link between autophagy and apoptosis. Both processes share common regulatory pathways and effector molecules. Both can exert an either positive or negative effect on each other and sometimes activation of both may coexist in the same tumor. However, the majority of studies supporting the autophagy-dependent cell death were performed in cells deficient in apoptosis (91), suggesting that autophagy represents only a non-canonical pathway leading to cell death. The exact regulatory mechanisms that influence the equilibrium between apoptosis and autophagy and ultimately determine cell fate, either cell death or survival, remain elusive.

The results of a few recent studies support the role of autophagy-dependent cell death in TC. Reversine, a synthetic purine analog has been shown to induce apoptosis and cell cycle arrest in differentiated and poorly differentiated TC both *in vitro* and *in vivo* in mouse xenograft models (92). Lu et al. (93) showed that reversine was also able to induce phagosome formation in WRO cells in a dose-dependent manner possibly through the Akt/mTOR/p70S6K pathway that was suppressed after exposure to reversine. Another argument to support the crosstalk between autophagy and apoptosis is provided by the studies on the antitumoral effects of statins. Zeybek et al. showed that in PTC and normal thyroid cell lines treatment with rosuvastatin, the statin inhibited the cell proliferation and induced cell death in a dose-dependent manner. In this study, autophagy was particularly activated in the BC-PAP cell line (BRAF V600E positive) at lower doses whereas at higher doses induction of apoptosis was predominant (94). Furthermore, Lopergolo et al. investigated whether the combination therapy with the RET-targeting tyrosine kinase inhibitor sunitinib and cisplatin can enhance apoptosis in MTC cell lines harboring the RET M918T oncogene and xenograft mouse model (95). The authors found that sunitinib induced a severe autophagosome accumulation and lysosomal dysfunction and cisplatin induced additional lysosomal leakage. Combination therapy resulted in more apoptotic cell death and a better response in xenograft mouse model with MTC.

The relation between cancer cell metabolism and its impact on cell survival, proliferation, and autophagy in TC has only scarcely been studied. In a recent study, Morani et al. have investigated the impact of PTEN deficiency and mutant p53 on glucose metabolism in two TC cell lines, the FTC133 (PTEN null, p53 mutated) and WRO (wild type p53 and PTEN). They have found that the FTC133 cell line that display impaired apoptosis and reduced autophagy due to the somatic mutations in PTEN and p53 were clearly more sensitive to glucose restriction than the WRO cells in which glucose deprivation was able to stimulate autophagy and therefore offer the cells an escape route to prolonged survival (96). This is clearly in line with the other studies showing that in apoptosis, deficient tumor cells that are depending for the increased energy requirements on an increased influx of glucose for glycolysis, maintaining an intact or an increased autophagy response is crucial for cell survival in conditions of nutrients deprivation. Defects in autophagy, such as those resulting from concomitant mutations in the PTEN or PI3K/Akt pathway render the cells more sensitive to glucose depletion. This phenomenon could potentially be explored for therapeutic tumor starvation strategies in TC.

## Autophagy and Tumor Invasion and Metastasis

The role of autophagy in tumor invasion and metastasis is less known. The epithelial-to-mesenchymal transition (EMT) represents an important step to invasion and metastasis. In this process that enables detachment of the cells from their natural environment tumor cells gain properties including enhanced plasticity that favors survival and metastasis and induce resistance to cytotoxic agents and radiotherapy. In this context, it has been found that autophagy is involved in the regulation of cell plasticity (97). In addition, it helps preventing anoikis (cell death after the cell has been detached from its extracellular matrix) and therefore promoting survival of cells that have been detached form their extracellular matrix (98). Furthermore, induction of autophagy in breast carcinoma cells has been shown to induce EMT and resistance to cytotoxic T-cell-mediated lysis, therefore targeting autophagy may potentially avoid the occurrence of this resistance (99). Interestingly in this context, Meng et al. reported recently that knockdown of BAG3, a protein involved in multicellular pathways, induces EMT in thyroid cells through activation of a E-cadherin suppressor, ZEB1 (100). Furthermore, BAG3 has been found to be involved in Beclin1-independent autophagy (non-canonical autophagy) (101). Li et al. found that Earle’s balanced salt solution (EBSS) starvation reduced the BAG3 expression *in vitro* in thyroid cells and that forced BAG3 expression suppressed autophagy and promoted the apoptosis in TC cells exposed to EBSS starvation, making the BAG3 an interesting potential target to be further explored for therapy (102). In another recent publication, Wang et al. have investigated the effects of long non-coding DNA (lncRNAs) on TC cells *in vitro* (77). The BRAF-activated lncRNAs (BANCR), which are novel regulators implicated in cancer biology, have also been shown to be overexpressed and involved in cell migration in melanoma. BANCR has also been shown to induce EMT in malignant tumors such as melanoma, colon cancer, and non-small cell lung cancer (103–105). The BANCR were overexpressed in four of the five investigated tissue samples of PTC and in IHH-4 PTC cell line compared to the normal thyroid tissue. Overexpressed BANCR markedly activated autophagy and inhibited apoptosis in the PTC cell line. Inhibition of autophagy abrogated the effects of BANCR on cell proliferation. In this study, there were no evident effects of BANCR on the cell migration in the TC cells. Therefore, although some studies suggest that autophagy may play a role in tumor invasion and metastasis, its place in the pathogenesis of TC is not well defined. Further studies to elucidate this process may uncover novel therapeutic strategies.

## Autophagy and Response to Therapy

Several studies have implicated autophagy in the tumor response and resistance to therapy with either ionizing radiation or other cytotoxic agents. It has been shown that treatment with ionizing radiation is associated with a strong induction of autophagy and this may contribute to cell survival and the acquired resistance to therapy in many cancer cells including breast, nasopharyngeal, pancreatic cancer, and malignant glioma (106–110). For this reason, inhibition of autophagy with chloroquine or hydroxychloroquine in combination with radiotherapy or chemotherapy has been investigated as a strategy to prevent therapy resistance and sensitize tumors to therapy. This, however, does not seem to be the case in TC. Yeung et al. published in 2007 the first observation on autophagy and TC in relation to therapy (111). In this study, the authors observed in a mouse xenograft model obtained with ARO and KAT4 ATC cell lines increased macroautophagy in the tumors from the mice treated with the antiangiogenic agent Combretastatin A4 phosphate. The authors suspected that this is likely explained by the induction of autophagy due to anoxia and starvation resulting from therapeutic vascular disruption in this model. A few years later, Lin et al. found that doxorubicine and radiation also induced autophagy in both human PTC samples and PTC cell lines (112). However inhibition of autophagy with 3-methyladenine (3 MA) decreased the radiosensitivity and chemosensitivity of the TC cells, suggesting that at least for the therapy resistant PTC stimulation of autophagy may be a potential therapeutic strategy. In line with this hypothesis, Lin et al. showed that stimulation of autophagy using the mTOR-inhibitor everolimus resulted in sensitization of PTC cells to both doxorubicin and radiation. Furthermore, the effects of everolimus and doxorubicin as radiosensitizers were synergistic. The authors suggest that the autophagy-dependent cell death, as detailed in the previous paragraph, mediated through Met kinase is the main mechanism behind these effects (113). Treatment with everolimus has also been shown to potentiate the activity of sorafenib and sunitinib, two MKI that target particularly the VEGF and the MAPK in MTC through induction of autophagy (114). Therefore, in contrast to other cancer, although treatment with both chemotherapy and radiotherapy were shown to induce autophagy in TC cells, the TC seem to profit from the synergistic activation of autophagy, possibly through mechanisms related to autophagic cell death to increase their sensitivity to the cytotoxic agents.

Radiotherapy and chemotherapy also induce oxidative stress and result in cell death. Interestingly, autophagy has been shown to play an important role in the response to chemotherapy of some tumors, in particular through influencing the immune response in the tumor microenvironment. It is known that treatment with chemotherapy can induce immunogenic cell death. In other words, as a result of treatment with chemotherapeutic agents, a succession of processes takes place resulting in apoptosis and necrosis. As a consequence, membrane exposure of calreticulin and release of other proteins such as HMGB1 and ATP takes place that in turn have the ability to attract and recruit antigen presenting cells such as macrophages and DC in the tumor microenvironment. The DCs engulf the damaged tumor cells (remnants) and through antigen-presentation activate T-cells that in turn exert an antitumoral effect on the remaining unaffected cancer cells, thus enhancing the antitumoral effect of the chemotherapeutic agent. In TC, it has been reported that treatment with selumetinib and IFN gamma increased the immunogenicity of PTC cell through increased HLA-ABC expression and was associated with increased T-cell activation and IL2 production by peripheral blood leukocytes co-cultured with treated PTC cell lines (115). Michaud et al. has shown in cell lines and mouse models that particularly the ATP release by the chemotherapy-induced dying tumor cells is highly dependent on the integrity of autophagy and is required to induce an antitumoral immunogenic response (116). In this study, autophagy-deficient tumors released significantly lower amounts of ATP and were not able to elicit antitumoral immunogenic responses in response to therapy with methotrexate. The immunogenicity of the autophagy-deficient cells could be restored when treated with ARL67156, an inhibitor of ecto-ATPases that increased the extracellular ATP concentrations and when treated with recombinant human IL-1β, whose production depends on the availability of ATP. Furthermore, when treated with methotrexate *in vivo* in immunocompetent mice, only the growth of autophagy-competent tumors could be reduced as compared to the autophagy-deficient tumors. In contrast, there was similar lack of response to treatment of these tumors in immunodeficient mice, regardless the autophagy status. This suggests that the immunogenic response is an essential contributor to the efficacy of chemotherapy and that this response is highly dependent on the integrity of the autophagy pathway.

Autophagy has also been linked to response to treatment with proteasome inhibitors. Autophagy is closely related to the ubiquitin-proteasomes system (UPS), the other main intracellular protein degradation pathway within the eukaryotic cells (117). Proteasome inhibitors, including the multipathway inhibitor bortezomib, are promising agents for treatment of highly aggressive cancer (118). Studies performed on several cancer cell lines demonstrated that autophagy inhibitors can synergize with the cytotoxic effects of the proteasome inhibitors (119–121). Based on these preclinical data, a number of trials investigating autophagy inhibition in combination with proteasome inhibitors have been initiated. In TC, Zhang et al. found that proteasome inhibitors induced a decrease in Beclin 1 expression (122). However, the exposure to proteasome inhibitors caused beclin 1-independent macroautophagic cytotoxic responses in TC cells. In this context, in TC cells Beclin 1 was able to potentiate the antitumoral effects of the proteasome inhibitors in an autophagy-independent manner via survivin and suppressin.

An important mechanism contributing to therapeutic resistance of cancer cells involves activation of pathways that confer stem cell-like characteristics onto cancer cells (123, 124). This process is strongly linked to EMT, which promotes cell survival and invasiveness, resulting in more aggressive tumor phenotype. Recent studies suggest a link between the EMT, stem cell-like characteristics, autophagy, and resistance to cytotoxic agents (97, 99). Hinterseher et al. reported a clear expression of hedgehog pathway signaling factors, which are involved in the survival of stem cells, in two ATC cell lines (Hth 74, C643) and in primary tumor samples (125). Moreover, treatment of these cell lines with the hedgehog inhibitor cyclopamine showed a time- and dose-dependent inhibition of cell growth. Activation of hedgehog pathway has also been reported in MTC (126). Inhibition of hedgehog pathway has been shown to activate autophagy (127). Therefore, exploring how modulation of autophagy in this context may help prevent therapeutic resistance is warranted.

Summarizing these data, modulation of autophagy has the potential to synergize and contribute to the effect of other cytotoxic treatments and reduce resistance to treatment, which should be further explored in the context of multimodality treatment in cancer and personalized medicine.

## Implications for Therapy

### Implications of targeting autophagy for thyroid cancer therapy: Preclinical studies

Genetic alterations in the PI3K/Akt/mTOR pathway cause an addiction to this pathway in TC cells and create sensitivity to inhibition by targeting players in this pathway, such as Akt and mTOR (128). Among these, mTOR is of particular interest due to its involvement in the control of cell proliferation. Inhibitors of mTOR such as rapamycin, everolimus, and temsirolimus have all been shown to inhibit tumor cell proliferation in several cellular models. Rapamycin effectively inhibits proliferation and induces apoptosis of SW579 cells. Furthermore, the invasive ability of SW579 cells decreased when treated with rapamycin. In contrast, no obvious changes were observed in the expression of Akt indicating that there might be a feedback loop effect by mTOR inhibition (129).

RAD001 (everolimus) is an orally bioavailable inhibitor of the mTOR pathway. Papewalis et al. demonstrated that RAD001 inhibits cell growth in ATC cell lines (130). Also temsirolimus, a specific mTOR inhibitor showed the potency of suppressing growth in several cell lines that harbored genetic alterations in the PI3K/Akt/mTOR pathway with IC_50_ (concentration at which 50% inhibition occurs) in the nanomolar range (128).

Mammalian target of rapamycin exists in two different complexes, mTORC1 and mTORC2, which could both be targeted by potential anti-cancer agents. Rapamycin inhibits the mTORC1 complex, but not the mTORC2 complex (131). The general view is that mTORC2 regulates Akt and, as a consequence of only targeting mTORC1 activity, it has been shown that drug resistance quickly develops due to compensatory activation of Akt (132, 133). Therefore, it seems essential to target both the mTOR complexes for desired anti-cancer effects (134). In the last few years, several mTOR ATP-competitive inhibitors have been reported acting upon mTOR in both complexes and showing a more complete anti-cancer activity in comparison with that of rapamycin and its derivatives (135). These new mTOR inhibitors provide a method to avoid the feedback up-regulation of p-AKT observed with mTORC1 inhibitors (136). For example, INK128, a dual mTORC1 and mTORC2 kinase inhibitor, inhibited growth at low nanomolar concentrations in cell lines and 5-week-old Tg-RET/PTC3 mice treated with 3 mg/kg for 2 weeks displayed a reduced proliferation index (136). Treatment with Torin2, another second-generation mTOR inhibitor, inhibited cell viability and induced caspase-dependent apoptosis via activation of mitochondrial apoptotic pathway in PTC cells. In addition, on PTC xenograft tumor growth in nude mice Torin2 treatment induces anti-cancer effects (137).

Another way to avoid the negative feedback loop in mTORC2 is to directly inhibit Akt. KP372-1, an Akt inhibitor, blocked signaling downstream of Akt in thyroid tumor cells, leading to inhibition of cell proliferation and increased apoptosis. The major advantage of KP372-1 over Wortmannin and LY294002 as PI3K inhibitors is its greater efficacy and the marked induction of apoptosis in cancer cell lines. This may be due to its targeting a central downstream molecule and also due to the potential for a number of processes to bypass effects at the level of PI3K (138). Moreover, *in vivo* use of LY294002 in mice has been associated with many adverse effects, including death (139). Similarly, Wortmannin has demonstrated hepatic and hematopoietic toxicity (140). Therefore, although Wortmannin and LY294002 inhibit the PI3K/Akt pathway, their drawbacks raise concerns about their suitability as leading candidates for further development as anti-cancer drug. However, despite its greater efficacy, a potential downside of Akt inhibitors is toxicity because of the importance of Akt signaling in many normal cellular processes such as insulin signaling, and the lack of selectivity of the current Akt inhibitors including KP372-1 to different Akt isoforms (138).

Over the years, several new and selective Akt inhibitors have been developed. MK2206 is a recently developed novel non-ATP-competitive allosteric Akt inhibitor. Liu et al. tested the therapeutic potential of MK2206 for TC using various TC cells with known genotypes in the PI3K/Akt pathway. They demonstrated its potent and efficacious inhibition of proliferation with IC_50_ values in the low micromolar range, mostly below or around 0.5 μm, of TC cells that harbored mutations in the PI3K/Akt pathway (141). Although Akt inhibitors show effect in different cell lines, they are less potent as a single-drug than mTOR inhibitors. A prior report demonstrated that the presence of genetic alterations of PTEN, PIK3CA, and Akt1 correlated well with sensitivity to an Akt inhibitor, but had weaker correlations with the sensitivity to an mTOR inhibitor (128). This discrepancy suggests that mTOR activity does not depend solely on PI3K/Akt activity and may explain the lower efficacy of Akt inhibitors in comparison with mTOR inhibitors.

### Implications of targeting autophagy for thyroid cancer therapy: Clinical studies

Novel small-molecule protein-KIs have shown promising results in clinical trials on thyroid cancer, including axitibib (142), sorafenib (143), motesanib (144), and pazopanib (145). These KI mainly target the RAS–RAF–MEK–ERK pathway and prevent angiogenesis by inhibiting vascular endothelial growth factor receptors. KIs that inhibit the PI3K/Akt/mTOR pathway are less common, but the rapamycin analog everolimus, has recently been shown to have potential in clinical trials.

Lim et al. (146) published the first clinical trial of the activity of everolimus in patients with progressive, locally advanced or RAI refractory TC including all histologic subtypes. The primary end point was disease control rate, defined as partial response and stable disease ≥12 weeks. The patients self-administered everolimus 10 mg orally once daily until unacceptable toxicity or disease progression was reached. Disease control was observed in 31 (81%) patients and the median PFS was 47 weeks in all patients. However, a confirmed objective response was observed in only two patients (5%). Another phase-II trial included patients with metastatic, incurable RAI refractory, and progressive TC who were treated with 10 mg everolimus orally, showed similar results (147). The primary endpoint in this study was PFS. Only one patient experienced a partial response, whereas 18 (55%) and 10 (30%) achieved stable disease lasting 6 and 12 months, respectively. Interestingly, activation of autophagy without markers of apoptosis was detected in three patients subjected to sequential biopsies. It was concluded that the activation of autophagy could account for the higher rate of disease stability.

As KIs are known to induce stable disease only on a temporary basis, several mechanisms contribute to clinical resistance. Carracedo et al. showed that mTORC1 inhibition can activate MAPK through a PI3K-dependent feedback loop in human cancer (148). Activation of a different prosurvival signaling pathway upon mTOR inhibition explains as to why the tumor shows an initial response followed by rapid progression. However, the majority of known mechanisms of clinical resistance involve secondary mutations in the target kinase. Such mutations have been described for *ABL, KIT, EGFR, ALK, BRAF, MEK, PDGFRA, FLT3*, and *ROS1* (149). Recently, evidence was obtained that acquired resistance to mTOR inhibition can occur through these mechanisms (150).

Autophagy inhibition using hydroxychloroquine and other autophagy regulators in combination with different therapeutic strategies has been evaluated in early-phase clinical trials on various types of tumors (151, 152). Chloroquine and hydroxychloroquine inhibit autophagy at a late stage by blocking lysosomal acidification resulting in an inability to digest its engulfed cargo. This leads to increased cytotoxicity in combination with several anti-cancer drugs in preclinical models. These results led to multiple early-phase clinical trials in humans, mainly with solid tumors (151, 152). Although targeting autophagy in cancer will provide new opportunities for drug development, more potent and specific inhibitors of autophagy are needed. Moreover, the effect of autophagy regulators in TC should be further evaluated.

### Implications of targeting autophagy for thyroid cancer therapy: Synergistic strategies

Several combination therapies of autophagy modulating agents have been examined for their efficacy to inhibit TC progression, invasiveness, and metastatic disease.

#### Combination therapy: multiple targets within the PI3K/Akt/mTOR pathway

BEZ235 is a dual PI3K/mTOR inhibitor that reduces PI3K and mTOR kinase activity by competitive binding to the ATP-binding cleft of these enzymes (153). BEZ235 effectively inhibited cell proliferation in eight TC cell lines originating from four major histological types (PTC, FTC, poorly differentiated TC, ATC) with relatively low median effect doses (<44 nmol/L). In addition to inhibiting cell cycle progression, BEZ235 caused apoptosis in two out of six cell lines. Daily treatment in mice with 8505-C xenograft tumors delayed tumor growth during the therapeutic period and significantly degraded caspase-3, indicating that this compound may induce apoptosis *in vivo* (154). However, reactivated tumor growth observed after discontinuation of BEZ235 suggests that prolonged treatment is necessary to maintain therapeutic effect. However, BEZ235 was not able to repress p-Akt in all cell lines, indicating that the compensatory activation of Akt by inhibiting mTORC1 may still play a role (148). The novel allosteric Akt inhibitor MK2206 could completely overrule the feedback activation of Akt from temsirolimus-induced mTOR suppression, and the two inhibitors synergistically inhibited TC cell growth in OCUT1 and K1 cell lines (141).

#### Combination therapy: concomitant targeting PI3K/Akt/mTOR and other pathways

The involvement of multiple signaling pathways in aggressive TC suggests that it may be necessary to target several pathways simultaneously for effective treatment. Jin et al. demonstrated over 60% growth inhibition with combined MEK and mTOR inhibition in 10 cell lines using the MEK inhibitor AZD6244 (ARRY-142886) and mTOR inhibitor rapamycin (155). Recently, a combination of AZD6244 and GDC0941, a novel PI3K inhibitor, led to synergistic inhibition of TC cells that harbored genetic alterations in MAPK and PI3K/AKT pathways. Exposure to the combination of the compounds caused DNA fragmentations, which indicates strong apoptosis (156). These proapoptotic effects are essential since cell death is fundamental for eradication and hence cure of cancer. Other combination therapy also includes blocking VEGFR. RAF265 is an ATP-competitive pan RAF-inhibitor that also inhibits VEGFR2. RAF265 and BEZ235 strongly inhibit tumor growth, both *in vitro* and *in vivo* (157). The tested drug combination resulted in profound G1–G0 arrest and was associated with consistent inhibition of targeted kinases. These preclinical studies provide further support that dual targeting of the MAPK and PI3K pathways is more effective than targeting pathways individually. Sorafenib is an oral KI with *in vitro* activity against multiple targets, including RAF, RET, VEGFR1, and VEGFR2. An ongoing phase-II trial (NCT01141309) assesses the combination of sorafenib (400 mg twice a day) and everolimus (10 mg daily) in progressive RAI-refractory TC, excluding ATC. The combination of sorafenib and everolimus shows promising results: partial response rates were higher than those reported for sorafenib as a single agent, with a partial response of 56% for non-medullary TC. However, several grade-4 adverse events occurred possibly related to the drug (158).

Other phase-II trials are investigating combination therapy of everolimus and pasireotide, a novel somatostatin analog, in adults with RAI refractory DTC (NCT01270321) and sorafenib combined with temsirolimus in patients with TC of follicular cell origin (e.g., PTC, FTC, Hurthle cell carcinoma) (NCT01025453). Furthermore, although not yet investigated in TC, PI3k/Akt/mTOR inhibitors can be combined with autophagy regulators: the PI3K–mTOR inhibitor NVP-BEZ235 synergized with chloroquine to induce apoptosis in glioma xenografts (159).

#### Combination therapy of PI3K/Akt/mTOR inhibitors with conventional radiotherapy and chemotherapy

As mentioned before, PI3K/Akt/mTOR inhibitors may sensitize TC cells to current therapy by, e.g., inducing redifferentiation, as has been demonstrated for mTOR inhibition (160). Furthermore, external radiotherapy and chemotherapy may be used in extensively dedifferentiated TC. Chemotherapy options include doxorubicin, docetaxel, or the combination of cisplatin with doxorubicin for ATC. Several studies showed the synergistic effect of mTOR inhibition with chemotherapeutic agents; RAD001 (everolimus) sensitized PTC to doxorubicin and external beam radiation in a synergistic fashion. Treatment with 20 nmol/L of RAD001 decreased the IC_50_ of doxorubicin by 48 and 38% in 8505-C and TPC-1 cells, respectively, and PTC cells treated with RAD001 had a significant improvement in sensitivity to various doses of radiation (113). Although not yet approved for treating ATC, the combination therapy of BEZ235 and paclitaxel consistently demonstrated synergistic effects against ATC *in vitro* (154). Interactions between BEZ235 and paclitaxel, irinotecan, and etoposide were determined by calculating the combination index by Chou–Talalay equation. Synergistic effects were identified for the combination of BEZ235 and paclitaxel in all used ATC lines. BEZ235 combined with irinotecan also enhanced therapeutic efficacy, particularly when more cells were affected. BEZ235 plus etoposide only slightly increased cytotoxicity.

Moreover, a phase I study of daily everolimus plus low-dose weekly cisplatin was performed in 30 patients with advanced solid tumors not curable by surgery or radiation therapy, including 7 patients with TC (161). The phase II recommended dose is everolimus 10 mg/day (days 1–21) and cisplatin 20 mg/m^2^ (days 1, 8, and 15) of a 28-day cycle, potentially leading to phase-II trials combining everolimus and cisplatin for TC treatment.

### Targeting autophagy modulators in thyroid cancer: A potential redifferentiation strategy

Redifferentiation of TC cells that restores the sensitivity of the tumor to RAI therapy is considered an important potential therapeutic approach. Multiple strategies have been investigated for their potential to induce redifferentiation of TC cells, ranging from non-specific modalities such as retinoic acid and histone modification agents to treatment with specific oncogene-guided KIs, including MAPK, MEK, mTOR, and Akt kinases. Of particular interest to TC, inhibition of mTOR was demonstrated to increase the capacity of physiological thyroid follicular cells to accumulate iodine (35). Also PI3K-inhibition induced sodium iodine symporter (NIS) expression in rat thyroid cells and human PTC (162). By redifferentiation of TC cells, sensitivity to RAI therapy could be restored. Ho et al. reported recently that a short course treatment with selumetinib, a MAPK kinase inhibitor, resulted in an increase of ^131^I uptake sufficient to enable RAI therapy in 12 of 20 patients (163). Moreover, a similar effect was observed *in vitro* using an mTOR inhibitor. Pretreatment with rapamycin resulted in three- to fivefold higher amounts of iodine accumulated in both BC-PAP (PTC, BRAF V600 mutated) and FTC133 (FTC, PTEN deficient) cell lines by restoring functional hNIS expression (160). This study provides exciting new findings suggesting that mTOR inhibition, potentially mediated by activation of autophagy, is a promising new target for adjunctive therapy to improve the efficacy of RAI treatment. It is indeed well described that autophagy is also involved in mechanisms that determine the differentiation status of TC cells; active autophagy contributes to maintaining the differentiation status of TC cells after malignant transformation. In contrast, class I PI3K and mTOR block the autophagy process and induce dedifferentiation of TC cells. These effects on the differentiation status of tumor cells are exerted at the level of the functionality of the iodine uptake machinery with NIS expression being one of the most prominently regulated proteins (35, 162). These findings indicate that autophagy is closely related to RAI sensitivity, which makes it a promising target to combat RAI resistance. By reasoning, since also starvation is a potent inducer of autophagy, patient starvation using a hypocaloric diet prior to RAI treatment to induce local autophagy activity in thyroid cancer cells might also increase the clinical response to RAI therapy.

## Conclusion and Perspectives

Remarkable progress in understanding the molecular pathogenesis of TC has been made in recent years. Significant knowledge has been accumulated on the role of fundamental signaling pathways, such as the MAPK and the PI3K/Akt/mTOR pathways. These pathways provide new targets for therapeutic agents. This review focused on novel KI targeting the PI3K/Akt/mTOR and autophagy pathway. Several new KI have been developed of which dual mTORC1 and mTORC2 kinase inhibitors show great potential in different models, whereas classical mTOR inhibitors have already been tested in clinical trials.

Notably, targeting the PI3K/Akt/mTOR pathway is more effective in TC cell lines with mutations in this pathway. PTEN loss of function was, for example, identified as a predictor of sensitivity to mTORC1 inhibition in several cancers (131). Therefore, the concept of personalized medicine can be perfectly applied to patients with TC considering the causal mutations in different pathways described in various types of tumors. The characterization of the mutational profile is critical in the design of treatment with either single agent or combinational therapy and has been demonstrated to lead to more effective treatment in pilot studies (164, 165). Consistent with the idea of personalized medicine are findings that combination therapy is more effective in RAI refractory tumors, usually harboring mutations in several pathways, than targeting pathways individually (154, 156). Ongoing trials combining KI will reveal their efficacy in the future.

Although the role of autophagy in various settings in TC needs to be further elucidated, autophagy regulators can be of great potential. Induction of autophagy can sensitize thyroid tumor cells to other therapies and plays a major role in the regulation of apoptosis. Combining novel KIs with other cytotoxic therapy is another possible implementation in the development of novel therapeutic strategies for TC, for example, the combination of everolimus and chemotherapy. Moreover, the mTOR inhibitor rapamycin has shown to increase the iodine uptake and may therefore be a promising new target for adjunctive therapy for improving the efficacy of RAI treatments. In the coming years, further developments in treatment options and selective application of available agents are to be expected that will provide novel approaches to more effectively treat TC.

## Author Contributions

All authors (RM, VK, TP, JS) had substantial contributions to the conception of paper, reviewing the literature, drafting the manuscript, and revising it critically for important intellectual content. They have all approved the final version of the manuscript.

## Conflict of Interest Statement

The authors declare that the research was conducted in the absence of any commercial or financial relationships that could be construed as a potential conflict of interest.
